# The Concept of an Epilepsy Brain Bank

**DOI:** 10.3389/fneur.2020.00833

**Published:** 2020-08-20

**Authors:** Lizbeth Hernandez-Ronquillo, Hajar Miranzadeh Mahabadi, Farzad Moien-Afshari, Adam Wu, Roland Auer, Viktor Zherebitskiy, Ron Borowsky, Marla Mickleborough, Richard Huntsman, Mirna Vrbancic, Francisco S. Cayabyab, Changiz Taghibiglou, Alexandra Carter, Jose F. Tellez-Zenteno

**Affiliations:** ^1^Saskatchewan Epilepsy Program, Division of Neurology, Department of Medicine, University of Saskatchewan, Saskatoon, SK, Canada; ^2^Department of Anatomy, Physiology and Pharmacology, College of Medicine University of Saskatchewan, Saskatoon, SK, Canada; ^3^Epilepsy Program, University of British Columbia, Vancouver, BC, Canada; ^4^Division of Neurosurgery, Department of Surgery, University of Saskatchewan, Saskatoon, SK, Canada; ^5^Department of Pathology and Laboratory Medicine, Royal University Hospital, Saskatchewan Health Region, University of Saskatchewan, Saskatoon, SK, Canada; ^6^Cognitive Neuroscience Laboratory, Department of Psychology, College of Arts and Science, University of Saskatchewan, Saskatoon, SK, Canada; ^7^Division of Pediatric Neurology, Department of Pediatrics, University of Saskatchewan, Saskatoon, SK, Canada; ^8^Department of Clinical Health Psychology, Ellis Hall, Royal University Hospital, Saskatoon, SK, Canada

**Keywords:** epilepsy, brain bank, human, neurodegenerative diseases, epilepsy surgery, clinical characterization

## Abstract

Epilepsy comprises more than 40 clinical syndromes affecting millions of patients and families worldwide. To decode the molecular and pathological framework of epilepsy researchers, need reliable human epilepsy and control brain samples. Brain bank organizations collecting and supplying well-documented clinically and pathophysiologically tissue specimens are important for high-quality neurophysiology and neuropharmacology studies for epilepsy and other neurological diseases. New development in molecular mechanism and new treatment methods for neurological disorders have evoked increased demands for human brain tissue. An epilepsy brain bank is a storage source for both the frozen samples as well as the formaldehyde fixed paraffin embedded (FFPE) tissue from epilepsy surgery resections. In 2014, the University of Saskatchewan have started collecting human epilepsy brain tissues for the first time in Canada. This review highlights the necessity and importance of Epilepsy Brain bank that provides unique access for research to valuable source of brain tissue and blood samples from epilepsy patients.

## Introduction

### History of Brain Banks in Canada

The pioneer of brain banking in Canada was Dr. Ali Rajput at the University of Saskatchewan. In 1968, Dr. Ali Rajput created the first brain bank in Canada dedicated only to patients with Parkinson's disease (PD) and other movement disorders. Development of the Bank led to numerous international and local contributions and critical breakthroughs in the modern treatment of PD, only possible because every case has the full documentation of ^1^ (1) detailed longitudinal clinical history, frozen tissue, and complete neuropathologic examination (2–6). The bank has more than 400 brains from patients with PD and other movement disorders and it shares samples with national and international qualified scientists for free (1).

In 1980, McGill University created the next brain bank in Canada. The bank is based at the Douglas Mental Health University Institute and has become one of the largest brain banks in the world. Currently, it houses more than 3,000 brains as well as a large relational database containing demographic, clinical and developmental histories from the donors^2^. The bank has brains from people who suffered from different neurodegenerative diseases such as PD and other dementias, schizophrenia, major depression, bipolar disorder, substance use disorder and patients who committed suicide. The brain bank at the McGill University is internationally recognized and receives annual requests from many neuroscientists from Canada and abroad.

In 1993, Dalhousie Medical School established the Maritime Brain Tissue Bank^3^. The bank houses more than 800 brains from donors in the Maritimes and the tissue is available for local, national and international researchers. The bank focuses on Alzheimer's disease (AD) dementia and non-Alzheimer dementia, and is in the process of expanding its facilities to include cases of spinal cord disease, Amyotrophic Lateral Sclerosis (ALS), epilepsy, PD, multiple sclerosis, spinal cord injury, stroke, and schizophrenia.

In 2014, the University of Saskatchewan created the first epilepsy brain bank in Canada^4^. This article reviews the concept of an epilepsy brain bank and the potential research that can be done with these samples. An epilepsy brain bank with living donors, such as the one established at the University of Saskatchewan, enables correlation of clinical information and investigations with findings in tissues. This article is divided into sections, the first explaining the work done in patients before epilepsy surgery and the potential correlations with potential research in the samples. Other subsequent sections point out the initial lines of research questions and priorities with the brain-banked tissue samples.

### Why a Brain Bank for Epilepsy?

Epilepsy comprises more than 40 clinical syndromes affecting 50 million people worldwide. People with epilepsy experience unprovoked recurring seizures. This is a disease that poses significant challenges for modern medicine, as ~36% of patients receiving anti-convulsant medication have inadequate seizure control (7). In addition, up to 50% of patients with epilepsy suffer from behavioral co-morbidities such as impaired cognition, anxiety, and depression, which can often be more debilitating for the patients than the seizures themselves (8). The median incidence rate of epilepsy in developed countries ranges from 25 to 50 per 100,000 people per year, whereas in developing countries it ranges from ~30–115 per 100,000 people per year due to the addition of acquired infectious epilepsy, such as neurocysticercosis (9). In Canada, the prevalence of epilepsy is 50 cases per 100,000 people (10).

Partial seizures account for up to 50–60% of epilepsy cases in adults (incidence and prevalence) and temporal lobe epilepsy (TLE) is the most common type of partial epilepsy (10). Approximately 36% of patients with epilepsy do not respond to medication but this percentage is even higher for patients with TLE, where 70% of patients typically do not benefit from current drug treatments (11). Years of uncontrolled epilepsy can lead to cognitive decline and psychiatric complications and increase the burden of epilepsy on society by reducing the likelihood that TLE patients will lead a productive and independent life. In addition, patients with TLE have a much higher mortality rate compared with the other population (12). Current drug treatments have low efficacy rates in patients with TLE, but a surgical treatment option does exist. A well-conducted randomized controlled trial has demonstrated that surgical management of TLE is superior to pharmacological management in patients with drug resistant epilepsy (13). Evidence also suggests that the results of epilepsy surgery are sustained over time, and that the surgery can provide innumerable benefits in patients including enhanced quality of life, decreased mortality, and enriched social networks.

### What Is an Epilepsy Brain Bank?

An epilepsy brain bank is a storage source for pieces of tissues from epilepsy surgery resections. The tissues are donated by living patients undergoing brain resection for the treatment of their medically refractory epilepsy. Brains are properly extracted, dissected and orderly stored either in formalin-fixed-paraffin-embedded (FFPE) blocks or cryopreserved in −80°C freezers. Control tissues can be obtained from brain surgeries in the same center from patients with other neurological conditions such as brain tumors, vascular malformations and other focal lesions. These patients may have associated seizures but also, they could be seizure-free being an adequate control for some studies. In addition to accepting donated control brain tissues we will seek other brain banks to find and receive suitable control brain tissues as well, for example normal tissue. Alternatively, the aspirated tissue from resection margins, if available, could also serve as the healthy control tissue for each patient's seizure focus.

### Why Is It Important to Develop Epilepsy Brain Banks in Canada and Elsewhere Around the World?

The discovery of the molecular underpinnings and potential treatments of human epilepsy based on new molecular targets is demonstrably lagging behind progress made in neurodegenerative diseases such as AD and PD (14), partly due to the lack of availability of properly stored human tissues for the research. In neurodegenerative diseases, post-mortem brains have helped advance research, however, as epilepsy patients are usually much younger, post-mortem brain donation will not represent this population at all. Tissue donation during active disease is a solution to this problem, so long as the excised and ethically approved stored brain tissue after epilepsy surgery does not compromise standard of care including classic pathology assessment of the tissue.

Many fundamental questions in epilepsy remain unanswered: why do some epilepsy patients fail to respond to antiepileptic drugs(AEDs)? Why, despite newly developed epilepsy drugs, has their efficacy not improved? Why do 30 to 40% of epilepsy patients never respond to AEDs despite new drugs appearing in the market every year? Are there any better targets for developing new AEDs? Is it possible to develop antiepileptogenic drugs as opposed to antiepileptic drugs? What are the mechanisms of neurodegeneration in the epileptic brain? Are there targets for drug development with ability to prevent neurodegeneration and memory impairment in patients with epilepsy?

As per current standard of care in most epilepsy centers, the brain tissue obtained from temporal lobectomies or other types of temporal resections is fixed in formaldehyde to undergo routine pathological analysis. No further research studies are usually performed on the specimen. In addition, formaldehyde-fixed tissue is not suitable for most advanced research methods such as molecular biology, proteomics, and genomics. It is extremely crucial to perform further studies on brain samples using advanced methods, such as bulk or single cell RNA sequencing (RNAseq), mass spectrometry and transcriptomic analysis, coupled with traditional techniques of molecular biology, biochemistry, and immunohistochemistry to answer important questions. For example, how does prolonged seizure damage hippocampal neurons, and what is the role of glutamate receptor expression, and specifically do anti-seizure medications that attenuate glutamate receptors overexcitation protect hippocampal neurons and preserve memory?

The initial step is saving fresh brain specimens for molecular biology/ biochemical studies with storage at −80°C in a freezer. Concurrently obtained blood samples from patients will also be banked for future epilepsy-related genetic studies. Our center has started saving the brain from epilepsy surgeries and we encourage other Canadian epilepsy centers to begin this process, as expanding the tissue source will provide a richer resource for sharing epileptic and control tissue that could be used to address the following: (1) To detect the chronic differences in regions of the brain prone to seizures using proteomics, immunohistochemistry, and molecular biology; (2) To identify biomarkers of refractory epileptic activity, the common abnormal pathways shared by many patients with temporal epilepsy will be used as biomarkers of refractory epileptic activity; (3) These biomarkers will be translated into therapeutic targets, which will be tested in animal models to determine efficacy prior to clinical trials; (4) Target binding of these compounds in humans at therapeutic doses will be assessed using Positron Emission Tomography (PET) scanning with specific ligands (e.g., 19F-Flurodeoxyglucose).

### The Advantage of Epilepsy Brain Bank Over Brain Bank for Neurodegenerative Diseases

There are several advantages:

- No large storage space is needed. In neurodegenerative diseases, brain donation is post mortem and therefore the whole brain tissue is stored (usually half frozen and half formalin fixed), however, in epilepsy brain bank a small piece of surgically dissected tissue is stored.
- In brain banks for neurodegenerative diseases, the tissue is harvested post-mortem and therefore, hypoxic-ischemic changes in the brain are unavoidable. However, in an epilepsy brain bank, the tissue can be processed and stored quite quickly after surgical excision with minimal chance of hypoxic-ischemic changes. In addition, in samples from an epilepsy brain bank the entire tissue is preserved evenly since the small pieces of tissue are rapidly frozen, resulting in the inner depth of the tissue being frozen almost instantaneously as the tissue surface, whereas the freezing procedure required for a brain hemisphere with degenerative conditions could take longer thereby adding increased risk of hypoxic/ischemic brain damage.
- The samples are a better representation of the population as they are usually harvested during ongoing, active disease in living patients as opposed to neurodegenerative samples that are usually harvested post mortem and much later in the course of the disease.

### Methodology of Developing an Epilepsy Brain Bank at the University of Saskatchewan

After applying for and obtaining human biomedical ethics approval (often the most time-consuming step), a reliable storage location for both the frozen samples as well as the formaldehyde fixed paraffin embedded (FFPE) tissue specimens has been established with funding support from institutional and provincial funding agencies (e.g., College of Medicine at University of Saskatchewan, and Saskatchewan Health Research Foundation). All samples were coded and kept in locked locations, with the decoding data and patient's clinical information stored on a password protected computer using reliable software. The freezer must be equipped with emergency power as well as an alarm system to indicate any temperature change. Tissue collection, processing and storage was a collaborative process involving an epilepsy surgeon, two epileptologists, research coordinator, and two neuropathologists. After specimen excision, the tissues are placed in saline solution in sterile plastic containers, labeled based on anatomical location, for transport to the neuropathology lab. Neuropathologists then confirm the anatomical locations and cut the samples into smaller pieces, selecting the required amount of tissue for clinical pathological staining and diagnosis, with the rest left for banking. Any portion of formalin fixed samples saved for clinical pathology but not ultimately used will also be later transferred to the brain bank for storage. Fresh pieces of tissue are slow frozen with isopropyl alcohol in cryostat Eppendorf tubes and then stored in the locked −80°C freezer. The location of tissue in the freezer (shelf #, location on the shelf), patient coding information and clinical data are then entered into secure software developed by the University of Saskatchewan information technology department, on a password protected computer located in the same locked room where the freezer is located. Only the two epileptologists who are the biomedical ethics applicants have the access keys to the tissue storage room. A sample of patient's blood is also stored in the same freezer for possible future analysis. The neurosurgical suctioning and/or ultrasonic aspiration supernatant if available can also be centrifuged and saved for use in proteomics experiments.

### Potential Correlation of fMRI Studies With Tissue

Functional magnetic resonance imaging (fMRI) is a non-invasive tool increasingly used for mapping eloquent cortex in patients prior to brain surgery. The most common clinical application of fMRI is for functional mapping of basic language (15, 16) and simple motor tasks (16). While many labs rely on silent reading, it has been shown that reading aloud serves to better activate regions of the language network in the temporal lobe (15), in particular using exception words that cannot be read phonetically (17, 18). In addition to the more common language and motor tasks, recent evidence has emphasized the value of mapping visual attention-shift networks which are important for reading (19). Furthermore, given the location of the temporal resection, memory and emotional processing are also of principal concern in TLE patients (16), including regions near the fusiform gyrus for emotional face processing and parahippocampal gyrus for memory and emotional place processing. Finally, people with epilepsy have heightened visual perceptual sensitivity, which has been associated with cortical hyperexcitability (20). Such hyperexcitability of visual cortex has been demonstrated via repetitive visual stimuli (21).

Patient's scans are taken during interictal periods allowing for a neurocognitive assessment of the state prior to surgery. Our pre-surgical planning includes fMRI localization of eloquent cortex comprising spoken language, motor, attention, memory, emotional face and place processing and visual cortical excitability. Our fMRI battery not only provides a neurocognitive assessment useful for pre-surgical planning but also for basic research. Images are collected using a 3 Tesla Siemens Skyra MRI and have been summarized elsewhere (15). During the scan, we will also be running diffusion-weighted tensor imaging (DTI) to noninvasively trace neuronal white matter tracts in the patients. The fMRI images are considered prior to and during the patient's surgery to assist in localization of function proximal to the resection line, particularly with respect to language.

### Pediatric Specimens

Epilepsy surgery has become an accepted treatment option for children with medically refractory or treatment-resistant epilepsy (10). Pediatric patients requiring epilepsy surgery have seizures caused by a wide range of pathologies particularly within the extra-temporal regions. The clinical outcome of epilepsy surgery in these patients is variable depending on many factors including the type and location of lesion resected (22). The development of an epilepsy brain bank that includes specimens procured from pediatric epilepsy surgery patients presents an exciting and unique opportunity to study the pathophysiological mechanisms underlying pediatric medically refractory epilepsy. The characteristic features exhibited by the pediatric epilepsy population can be correlated and further explored with the brain tissue bank, as follows: (A) Developmental brain lesions such as cortical dysplasia and developmental brain tumors including gangliogloma (GG) and dysembryoplastic neuroepithelial tumors (DNT) are common causes of medically refractory epilepsy in children. Recent advances in the field of neuropathology have allowed, by examination of surgically resected tissue, a greater understanding of the pathophysiological mechanisms underlying the epileptogenesis of these lesions (23). (B) Although many different pathophysiological mechanisms have been proposed, much remains to be learned about the epileptogenesis of these lesions. (C) Enhancing our knowledge in this field has the potential to allow for the development of targeted anti-epileptic medical therapies in children and adults with developmental brain lesions. (D) Children can present with other causes of medically refractory epilepsy that is amenable to surgical treatment. This includes Rasmussen's Encephalitis, which is an inflammatory disorder that has a predilection in childhood (24). While much has been learned about the pathophysiology of Rasmussen's Encephalitis, little is known regarding the underlying cause of the inflammatory processes and the susceptibility to this disease in patients. Therefore, the epilepsy brain bank could provide a valuable tool to probe deeper into the mechanisms of medically refractory epilepsy in these pediatric patients.

### Surgical Techniques for Preservation of Brain

*En bloc* resection techniques are used whenever possible to maximize preservation of brain tissue. Once the epileptic focus is identified as a target for resection, the margins of resection are planned along anatomic borders. If the target gyrus is surrounded by non-eloquent brain, trans gyral resection through neighboring gyri allows for *en bloc* removal of the seizure focus along with a margin of normal brain. The trans gyral approach has the additional advantage of tending to result in less bleeding compared to trans sulcal and intralesional approaches. If neighboring gyri are eloquent and need to be preserved, the target gyrus can be isolated and removed trans sulcally. Sub-pial dissection along gyral borders helps preserve the integrity of neighboring gyral cortex, as well as the any vasculature within the sulci (25, 26). More complex resections may be planned as step-wise piecemeal removals of defined anatomic sections. For example, when performing a temporal lobectomy, if the patient's anatomy suggests that resection in a single bloc becomes problematic, the resection can be accomplished stepwise, first by removing the cortical plug in one block, exposing the temporal horn of the ventricle and the hippocampal head and body, followed by removal of the mesial structures including the hippocampal head as a second block, using subpial dissection to separate the hippocampus and uncus from the arachnoid of the underlying cistern below. This is then followed by removal of any remaining temporal tip tissue as a third bloc, and each of these blocks can be sent as separate specimens with their cytoarchitecture preserved. Intralesional aspiration is avoided whenever possible, and the use of ultrasonic aspirators is limited to dissection at the resection margins, if needed (25, 26).

**Figure 1:**
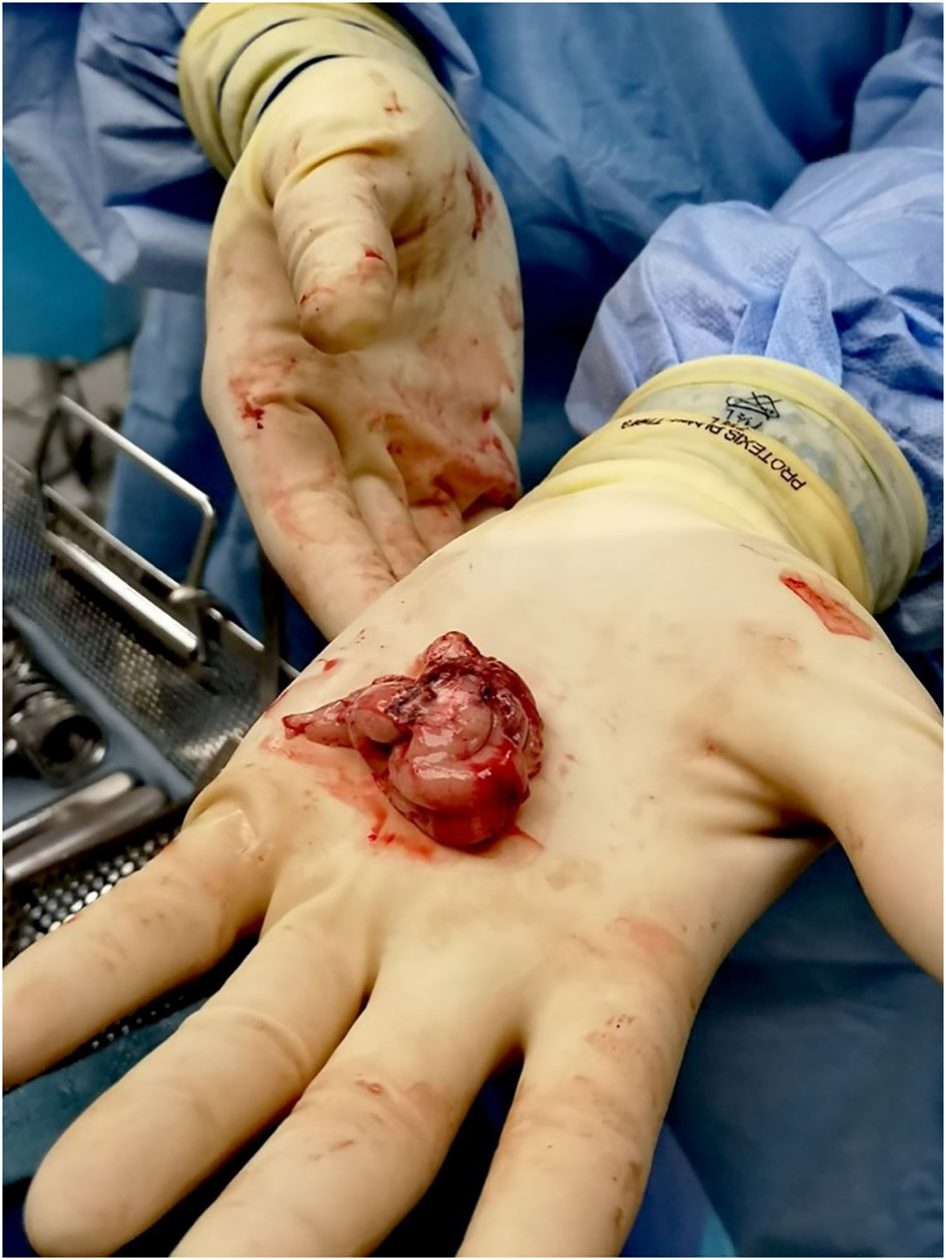
En bloc resection techniques are used whenever possible to maximize preservation of brain tissue. Once the epileptic focus is identified as a target for resection, the margins of resection are planned along anatomic borders. The figure shows one of our patients who had a standard temporal lobectomy.

In addition to maximally preserving brain specimens for research purposes, *en bloc* resection can provide clinical benefits including more specimens for pathological diagnoses, reduction of surgical blood loss, and, in the case of lesional resections suspected to be neoplastic, minimization of spillage of potentially neoplastic cells into the resection cavity as well as along margins of normal tissue around the resected suspected neoplasm where possible. Figure 1 shows a bloc resection of the temporal region (25, 26).

### Neuropsychology and Epilepsy Brain Bank

In individuals with epilepsy, disturbances in cognitive functioning, memory and language in particular, are frequent complaints and clinical findings, which can be very distressing or disabling. There are a number of common causes of epilepsy that have identifiable structural change in the affected brain region, but in the majority of epilepsy cases the cause is not known. A further complication is that some patients do not respond to antiepileptic drugs and their seizures remain intractable. Ongoing seizure activity in addition to antiepileptic load also has a negative impact on cognitive functioning. Slightly over half of seizures in adults are complex partial type and about 80% of these seizures originate in the temporal lobe. Resection of the temporal lobe is sometimes the best course of the treatment and allows for a closer examination of the affected tissue. This resected tissue has the potential to be very informative in a number of domains of investigation. Accordingly, all potential brain bank participants will be administered a comprehensive neuropsychological test battery that will include clinical measures of intelligence, language, visuoperceptual ability, immediate and delayed verbal and visual memory, executive functioning, fine motor speed and dexterity, psychomotor speed and cognitive flexibility as well as psychological well-being. These cognitive domains were selected in order to provide representative coverage of the major domains of higher cognitive functioning focusing on measures that are commonly used in clinical practice as well as providing an assessment of psychosocial adjustment. These same measures will be re-administered to brain bank participants post resection and will allow for examination and correlation of participant demographic factors, clinical characteristics, cognitive functioning and neuropathology to assist in better understanding the mechanisms underlying intractable temporal lobe epilepsy.

### Brain Bank and Neurophysiology

The epilepsy brain bank has been essential to supply the well-documented tissues to study the functional alteration and mechanism underlying the pathogenesis of epilepsy. Recent findings in neurophysiology have led to increased demands for postmortem human brain tissue. Advances in the fields of genomics and proteomics offer new ways to study the pathophysiology of neurological diseases. Immunohistochemical and electron microscopic analyses show that cortical slices originating from temporal lobectomy in temporal lobe epilepsy persist well in culture for about 11 days without any substantial changes. These collected tissues have intact neuropil, endothelial cells, basal lamina, and myelinating and non-myelinating axons. Moreover, mitotic bodies in tumor slices verify cell proliferation occurring in these cells that pave the way for studying cell composition, migration, progression and efficient pharmacological therapies for brain tumor.

Studies involving post mortem brain tissue may lead to improved understanding of the structural and molecular neuropathology (27) and further elucidation of molecular mechanisms of epilepsy (28). However, recent studies using antemortem tissue verify that epileptiform activity in temporal lobe and hippocampal slices can indeed be preserved and recorded *in vitro* (29, 30). The normal electrophysiological activity recorded from epileptic slice culture appears to be preserved for several weeks (31). Furthermore, the result of interictal-like activity from subiculum of human acute temporal lobe slice cultures shows the same pattern as intracranial electroencephalography (EEG) recording from the same patient with temporal lobe epilepsy (32). Also optogenetic stimulation has been employed to probe network mechanisms in human cortical and hippocampal circuits, suggesting proof-of-concept application of this technique on human brain tissue which could accelerate the future development of anti-epileptic drugs using this technique (33).

### Brain Bank and Neuropharmacology

In order to prevent, treat and cure the epilepsies, researchers need to determine what causes altered neuronal excitability that triggers the abnormal firing patterns during epileptic seizures. Epileptogenesis is a process of initial brain-damaging insult that leads to a cascade of molecular and cellular alterations. Cellular changes include neurogenesis, neurodegeneration, axonal and myelin injury, dendritic remodeling, blood brain barrier damage, alterations in extracellular matrix composition and possible aggregation of intracellular or secreted materials. Discovery of these mechanisms has the potential to yield new anti-seizure therapeutic targets.

Of all candidate mechanisms, one is currently in clinical trials, namely the inhibitor of mTOR (mammalian target of rapamycin) signaling Everolimus (in progress phase 2 trial for cortical hyperexcitability in tuberous sclerosis complex (TSC) and focal cortical dysplasia (FCD) (ClinicalTrials.gov Identifier: NCT02451696); a completed phase 3 trial with patients with TSC who have refractory partial-onset seizures (ClinicalTrials.gov Identifier: NCT01713946). mTOR hyperactivation signaling in epilepsy makes it an efficient target for therapeutic intervention and have driven the significant effort to pharmacologically target this pathway (34). Other potential targets include inhibition of cytokines interleukin-1β, interleukin-6, and interleukin-10 (35, 36). Interleukin-1β (IL-1β), a classically proinflammatory mediator of acute pathogenesis after injury, has been identified to initiate and aggravate seizure activity in epilepsy (37, 38). Blocking IL-1β synthesis or inhibiting signaling through IL-1 receptor can improve seizure severity and incidence (37). An inhibitor of IL-1β synthesis is being studied in patient with treatment-resistant epilepsy.

Other areas of study in epileptogenesis include plasma membrane proteins that are involved in neuronal communication by the passage of ions, such as sodium, calcium, chloride and potassium through these protein channels. A disruption in any of these processes could be involved in epilepsy. The prominent role of voltage-gated ion channels, glutamatergic receptors, and GABA receptors in epileptogenesis as a molecular target in the development of novel anti-epileptic drug has been reviewed elsewhere (39). Voltage-gated Na^+^ channel inhibition is the mechanism of action of phenytoin, carbamazepine and lamotrigine, and many other classic and novel anti-epileptic drugs. Retigabine (a neuronal KCNQ/Kv7 K^+^ channel opener), ethosuximide (a T-type Ca^+^ channel blocker), benzodiazepines [a positive allosteric modulator of GABA (A) receptor, promoting increased inhibitory neurotransmission], and felbamate/topiramate [having mixed actions: GABA (A) receptor agonist, sodium channel blocker, and NMDA receptor antagonist] are current therapeutic approaches for epilepsy (39). The knowledge of the role of ion channels from human brain samples is allowing the design of new and more exclusive therapeutic strategies (40).

Moreover, disruption of the blood-brain barrier could be one of the major causes in seizure pathogenesis. Leakage of blood derived proteins into fluid surrounding the brain, leads to hyperactivity of neurons in the area of the brain surrounding the leakage. During epileptogenesis, an increase in IL-1β and IL-1 receptor was found at the time of blood brain barrier damage (41).

Glial cells are non-neuronal cells with important supportive role in the brain. Astrocytes are a type of glial cell that are involved in the redistribution of elevated K^+^ and transmitter concentration during neuronal activity. Investigation of specimens from patients with pharmaco-resistant temporal lobe epilepsy and epilepsy models revealed changes in expression, localization, and function of astroglial K^+^ (42).

Recent findings reveal that ceftriaxone, an antibiotic that improves the housekeeping role of astrocytes, has been shown to decrease seizure frequency (43).

The relationship of immune system to the development of certain forms of epilepsy has been studied (44). In aggressive forms of epilepsy, antibodies may decrease the function of brain receptors, disrupting the neuronal function. Findings from early-stage clinical trials suggest that strategies aimed at adjusting the body's immune system may provide a means of treating these otherwise untreatable forms of epilepsy.

Brain bank collections that gather and provide standard samples clinically and pathologically are beneficial in the progress of advanced neurological research. Without brain banks facilitating the process of getting human brain tissues, it is more difficult for many neuroscientists to do research.

In previous studies, animal models were the most important source of sample to replicate human neurological diseases, although these models in some cases could not replicate completely the biochemical and cellular mechanisms involved in human neurological disorders because of the complexity of human diseases. Thirty-three percentages of patients with Temporal Lobe Epilepsy (TLE), the most common form of epilepsy, do not response to current antiepileptic drugs (AEDs). The AEDs used for non-epileptic disorders, such as rapamycin, everolimus, celecoxib, bumetanide, amiloride, resveratrol, and losartan have been studied in animal models of epilepsy with good outcomes (45). Losartan that is an angiotensin II type 1 receptor (AT1) antagonist has appeared as an anticonvulsant and anti-epileptogenic agent in animal models. Furthermore, the neuroprotective effects of this drug have been studied in animal models with Post-Traumatic Epilepsy (PTE) (46), kainic acid-induced status epilepticus (47), seizure in animal model with co-morbid hypertension and epilepsy (48), pentylenetetrazol (PTZ)-kindled seizures (49). In spite of the suppressive role of losartan in different kinds of animal seizure model, it failed to exert effect on epileptiform activity in human brain slices from patients with pharmaco-resistant TLE (50).

Brain banks combined with advanced modern neurobiological, proteomics and transcriptomic analyses can contribute to finding novel therapeutic anti-epileptic key targets directed by neuroscientists and pharmaceutical companies. The number of brain banks established worldwide has increased dramatically within the last decade, and this reflects an increased collaboration and engaging relationship between tissue donors, clinicians, neuropathologists, and neuroscientists. Epilepsy brain banks are confronting a crucial need for a consensus on the clinical and neuropathological diagnostic criteria for epilepsy that will make their samples appropriate for high-quality research. The identification and validation of biomarkers in both *in vivo* samples and autopsy cases have produced new attitudes toward neurological disorders. Well-documented autopsy material has been applied to study the human brain proteome in neurological diseases and in normal aging (51–54). The identification of altered genes in the human brain proteome in the early stages of the progress of epileptic brain diseases can provide valuable insight into potential biomarkers or novel targets for epilepsy that can be further developed that ultimately helps improve patient diagnosis and treatment.

Brain banks have used analysis of the membrane and soluble-fractions to identify perturbed genes in brain proteomes in neurological disorders. The manipulation of island-clustering analysis (55) following gel electrophoresis facilitates these comparisons between membrane and soluble protein fractions; this method is also capable of identifying and recognizing the biomarker proteins in neurological disorders and aging. Moreover, brain banked tissue samples can also be a valuable tool in identifying and confirming the global changes in gene expression and proteome dynamics in epileptic brains. Large-scale protein-protein interaction maps and hub genes generated from proteomics methods have recently been demonstrated to provide insight into the possible perturbed multi-protein complexes and genetic interactions in neuronal cells before and after differentiation (6). Whole cell, nuclear, cytosolic or extensive biochemical fractionation with in-depth mass spectrometry profiling could reveal widespread changes in the protein-protein interaction networks and hub genes that may facilitate our knowledge of the molecular mechanisms underlying different forms of epilepsy, which has the potential to improve the diagnosis and treatment of epilepsy.

Longitudinal clinical data can also be correlated with the proteomic findings and neuropathological correlates. During the 2011 Human Proteome Organization meeting, a decision was taken to launch the international, multidisciplinary Human Brain Proteome Atlas Project. The main goal of the plan was to analyze the proteomes of typical areas within the healthy human brain during normal aging, and to compare these with the proteomes of similar zones in subjects with specific diseases, such as epilepsy. This human proteome project and the proliferation of brain tissue banks worldwide will accelerate the identification of key molecules and pathways involved in epilepsy, and will also facilitate the knowledge and clinical translation of the proteomics findings generated by robust, advanced methods of high-throughput mass spectrometry and integrated bioinformatics computational analysis. Studies of the molecular and biochemical underpinnings of brain diseases using post-mortem brain tissue have inherent limitations (e.g., hypoxic brain tissue damage, possible degradation of native protein-protein interactions and RNA degradation), therefore, it is crucial to have a readily available ante-mortem brain tissue bank to generate protein-protein interaction network map that most resembles the native interactions occurring in the healthy or diseased brains (55–61).

In the brains of affected individuals, peptide and protein patterns are complex, and new molecular tools, such as MALDI-M, are needed to characterize these patterns and to identify specific proteins involved in neurological diseases such as epilepsy. MALDI-MS can be used to generate peptide and protein profiles of brain-tissue sections, and also enables the localization of neuropeptides and proteins in much smaller areas. These proteins at a high spatial resolution with markers of cellular components can document the localization patterns of molecular entities implicated in epileptic brain at the subcellular level (51–54). The advantages of having a readily available brain bank for studying these molecular markers for epilepsy can be significant in providing new information about the molecular nature of the patient's epilepsy, and with informed new knowledge and the recognition of a rational therapeutic goal, the patient can feel empowered and engaged in directing the type of research that can be pursued with the patient's own epilepsy surgery brain-banked tissue sample.

In recent years, some studies have yielded substantial progress in the classification of genetic mutations involved in different forms of epilepsies (62–64). Some types of epilepsy are associated with mutations in ion channels and ligand-gated ionotropic receptors (65, 66) Family members affected by certain epilepsy syndromes can undergo genetic testing to find possible underlying genetic mutations. The functional consequences of these mutations can be studied in transgenic animal models bearing the gene mutation, and this would then allow potential therapies to be introduced to treat the epilepsy phenotype. Ultimately the identification of a genetic basis for a type of epilepsy could improve the care and medical management of patients with epilepsy. Also armed with the knowledge of an existing family history of a heritable epilepsy-related gene mutation, the affected family members are informed of any precipitating factors that can increase their risk of having epilepsy. Therefore, close collaboration among the basic scientists and clinicians, and the generosity of patients in donating their brain tissue for brain banking will lead to investigations that will minimize the effects of many risk factors, including genetic basis, among large populations of people affected by the epilepsies. There is consensus that current brain-banked tissue samples should be accompanied by clinical and neuropathological diagnostic criteria to facilitate the clinical translation of high-quality research.

### Adenosine-Based Therapeutic Targets and Epilepsy Brain Bank

The development of novel neuroprotective therapies for patients suffering from pharmaco-resistant Temporal Lobe Epilepsy (TLE) is urgently needed; therefore, the human epilepsy brain bank will be utilized to confirm pre-clinical data gathered using animal models for TLE. Adenosine, an endogenous nucleoside that acts as a neuromodulator in the brain, plays an integral role in the pathophysiological etiology of TLE, where adenosine-based treatment strategies have proven to be efficacious. Increasing hippocampal adenosine signaling has been found to have anti-epileptiform actions through adenosine A1 receptors (A1Rs), inhibitory G protein coupled receptors that can induce profound synaptic depression (67). A1R activation has also been shown to be neuroprotective against excitotoxic neurodegeneration in the hippocampus of TLE animal models (68). There is continued interest in the cellular mechanisms by which adenosine A1R activation reduces epileptiform activity, as well as in identifying pharmacological targets involved in the actions of adenosine for neuroprotection.

Previous and ongoing investigations have demonstrated the involvement of adenosine A1 and A2A (A2AR) receptors in the pathophysiology of neurodegeneration in animal stroke models, which display post-stroke depression, cognitive dysfunction and increased post-stroke seizures (2, 69). An important physical and functional interaction between A1Rs and glutamate AMPA receptors (AMPARs) in rat hippocampus contributes to the robust AMPAR endocytosis and persistent synaptic depression observed after prolonged A1R activation or during hypoxic insult (69, 70). This functional and physical interaction between A1R and AMPAR in hippocampus underlies hippocampal neurodegeneration (70). Current investigations are aimed at further characterizing how prolonged A1R stimulation leads to altered compositions of AMPA receptor subunits (i.e., resulting in the over-expression of the calcium-permeable GluA1 AMPA receptor subunits) and altered surface expression of adenosine A2A receptors that could both promote increased hippocampal neuronal excitability and consequently generate seizures in our animal stroke models. Additionally, the equilibrative nucleoside transporters (ENTs) are passive nucleoside transporters that allow adenosine transport across cell membranes, and treatment with selective ENT antagonists has been shown to reduce neuronal excitability in animal models of TLE, similarly to A1R agonist treatment (71). Cannabidiol, a recently identified neuroprotective agent, has also been shown to inhibit ENTs (72), and we are currently investigating its potential as a neuroprotectant in the hippocampus by modulating extracellular adenosine levels.

In preliminary studies, elucidation of the signal transduction pathways activated by A1R stimulation in the hippocampus showed evidence of modulation of the expression, localization, and function of ionic channels, including the hyperpolarization-activated cyclic nucleotide-gated (HCN) channels (HCN1, HCN2, and HCN4) and the HERG potassium channels (Kv11.1). Importantly, key alterations in these channels have been shown to underlie some forms of epilepsy (73). These modifications in both HCN and HERG channels are suggested to contribute to the acquisition of recurrent spontaneous seizures and initiation of epileptiform activity, to promote the subsequent propagation of seizure activity, and to increase and induce the state of dendritic hyper-excitability in dentate gyrus granule and/or CA1 pyramidal hippocampal neurons to generate the characteristic hyper-excitability within hippocampal circuits in TLE. With the use of the human epilepsy brain bank to discover new genetic mutations, it is plausible to suggest in future studies whether genetic loss-of-function mutations in the voltage-gated HERG and HCN channels, ENTs and A1Rs exist to modulate neuronal excitability and induce seizures in patients.

### Importance of Epilepsy Brain Bank in the Development of Treatment Strategies

More than 50 million people worldwide suffer from epilepsy and about one third of these patients are considered resistant to pharmacotherapy, despite the availability of more than 20 anti-epileptic drug choices. In addition to incurring a higher drug cost to treat epilepsy, the consequence of drug resistant epilepsy in these patients is much more severe as judged by a four to seven-fold increase in mortality rates in this group compared to drug-responsive epileptic patients (7, 74, 75). Therefore, new classes of anti-epileptic medications are urgently needed to alleviate suffering of those individuals not responding to the currently available therapeutic agents. One of the major resources in the discovery of new therapeutic agents is repurposing or repositioning drugs from other therapeutic fields with potential beneficial effects in epilepsy. Repurposing of existing drugs is an attractive, cost effective, and timely drug development strategy considering the fact that drug candidates have been already tested and evaluated in terms of safety, toxicity and side effects in patients with different diseases (76). In fact, it usually takes more than 10 years and a heavy, as well as risky, investment to bring an entirely novel chemical or biological therapeutic agent from bench to clinic. In addition to utilizing a high-throughput methodology, having clear research objectives and a thorough understanding of pharmacological mechanisms of action and signaling pathways contributing to epileptogenesis is an important necessity. Although drug repositioning in CNS disorders is an active area of research and discovery for neurodegenerative diseases such as AD and PD, repurposing a wide variety of drugs, ranging from anti-inflammatory agents to neurotrophic factors, in treatment of epilepsy has recently gained considerable momentum and attention (77, 78). Tissues collected and preserved by epilepsy brain banks will serve well for the drug repositioning research. Analyzing signaling molecules affected in resected specimens will provide a better understanding of pathways pathologically impacted, which in turn will assist selecting appropriate drug candidates for the epilepsy drug repurposing study. Moreover, information collected from TLE post-operation samples will enable us to screen the novel target molecules and signaling pathways involved in the pathogenesis of epilepsy.

In recent years, studies on epilepsy animal models and post-surgical TLE specimens resulted in the discovery of several novel therapeutic targets (e.g., resveratrol, cyclooxygenase-2inhibitors, erythropoietin, and HMG-CoA reductase inhibitors) and signaling pathways including the potential involvement of interleukin 1β, mammalian target of rapamycin (mTOR), nuclear erythroid-2-related factor 2 (Nrf2), sphingosine 1-phosphate receptors, and peroxisome proliferator-activated receptors (PPARs). Although these discoveries are promising and considered a great step forward in combating epilepsy, they are still far away from patients' medicine cabinets. More research and studies are needed to further confirm and validate previous observations as well as revealing as yet unidentified epilepsy-related signaling pathways and entities. Indeed, the epilepsy brain bank will be a pivotal establishment for this purpose.

The main aim of this article is to help to stablish and spread the concept of an epilepsy brain bank. We believe that more institutions around the world should be interested in having epilepsy brain banks, especially in centers with active neurosurgical and epilepsy surgery programs. The epilepsy brain bank is also an opportunity for national and international collaborations in order to share tissue but also to share research ideas and technology to analyze the samples. Our article also explores the benefits of correlating clinical information of patients with the observations obtained from the human tissue. Once we have reached a large number of samples in our bank, we will work in the internationalization of our bank to collaborate with other centers.

## Author Contributions

LH-R, JT-Z, and FM-A develop the idea of the epilepsy brain bank. JT-Z and LH-R are the holders of the bank in the University of Saskatchewan. HM collaborated to write the whole document. AW wrote the section related with surgical techniques. RA and VZ wrote the section on neuropathology. RB and MM wrote the section of imaging. RH wrote the section on pediatric neurology. MV wrote the section neuropsychology. FC and CT wrote the sections on physiology and pharmachology. AC reviews the whole document. All authors contributed to the article and approved the submitted version.

## Conflict of Interest

The authors declare that the research was conducted in the absence of any commercial or financial relationships that could be construed as a potential conflict of interest.

## Footnotes

^1^State of Rural Canada. (2020). http://sorc.crrf.ca/saskatchewan/ (accessed April 2020).

^2^The Douglas Bell Canada Brain Bank (2020). http://douglasbrainbank.ca/ (accessed April 2020).

^3^Dalhouse University. (2020). https://medicine.dal.ca/research-dal-med/facilities/cores/maritime-brain-tissue-bank.html (accessed April 2020).

^4^University of Saskatchewan. (2020). https://medicine.usask.ca/news/2015/com-researchers-breaking-ground-with-epilepsy-brain-bank.php (accessed April 2020).
